# STING-driven activation of T cells: relevance for the adoptive cell therapy of cancer

**DOI:** 10.15698/cst2023.11.291

**Published:** 2023-11-14

**Authors:** Fabian Richter, Christophe Paget, Lionel Apetoh

**Affiliations:** 1 Centre d'Étude des Pathologies Respiratoires, U1100, INSERM, Tours, France.; 2 Faculté de Médecine, Université de Tours, Tours, France.; 3 Brown Center for Immunotherapy, Indiana University Melvin and Bren Simon Comprehensive Cancer Center, Indiana University School of Medicine, Indianapolis, IN 46202, USA.

**Keywords:** Adoptive T cell therapy, STING, T cells, cancer, immunomodulation

## Abstract

Adoptive cell therapy (ACT) can successfully treat hematopoietic cancers but lacks efficacy against solid tumors. This is due to insufficient T cell infiltration, high tumor heterogeneity, frequent antigen loss with subsequent tumor escape, and the immunosuppressive tumor microenvironment (TME). Alternative methods to boost the anticancer efficacy of adoptively transferred cells are actively pursued. Among adjuvants that are utilized to stimulate anticancer immune responses, ligands of the stimulator of interferon genes (STING) pathway have received increasing attention. STING activation can trigger dendritic cell (DC) activation and endogenous immune responses, thereby preventing tumor escape. Activation of the STING pathway in the context of ACT was accordingly associated with improved T cell trafficking and persistence in the TME combined with the reduced presence of immunosuppressive cells. Recent findings also suggest cell-intrinsic effects of STING ligands on T cells. Activation of the STING signaling pathway was in this regard shown to enhance effector functions of CD4^+^ and CD8^+^ T cells, suggesting that the STING signaling could be exploited to harness T cell anticancer functions. In this review, we will discuss how the STING signaling can be used to enhance the anticancer efficacy of ACT.

## THE RELEVANCE OF ADOPTIVE T CELL THERAPY IN CANCER

In 2018, Tasuku Honjo and James Allison were awarded the Nobel Prize for their research on immune checkpoint inhibitors (ICI). They found that inhibition of the suppressive molecules PD-1 and CTLA-4 enhanced the ability of the immune system to eliminate cancer cells [1]. Immune checkpoints are expressed on activated immune cells and serve to maintain self-tolerance and regulate immune responses. Tumor-induced engagement of these immune checkpoints is considered as a resistance mechanism that limits T cell activation [2]. Numerous anti-PD-1 and anti-CTLA-4 therapies were developed and showed unparalleled survival rates for patients with non-small cell lung cancer, renal cell carcinoma, and melanoma [3]. Although these treatments are now FDA-approved for many cancer types, a significant number of patients suffers from immune-related adverse effects or acquired resistance [4]. Especially in weakly immunogenic cancers, some patients cannot benefit from ICIs, highlighting the importance of further progress in this research area and the need for alternative therapeutic approaches [5]. One other treatment avenue with proven clinical success is adoptive cell therapy (ACT). In ACT, autologous immune cells are amplified ex-vivo, modified, and subsequently transferred back into the patient [6]. It has been successfully demonstrated that ICI and ACT can be combined [7, 8], possibly providing additional benefits [9]. Some metastatic melanoma patients in whom ICI showed no therapeutic effect were successfully treated with ACT, suggesting that ACT can be used when ICIs fail [10–13].

Three main T cell-based approaches are currently considered for ACT, including the use of tumor-infiltrating lymphocytes (TILs), antigen-specific T cells equipped with a specific T cell receptor (TCR), and chimeric antigen receptor T cells (CAR-T) [14]. TCR-based ACT does not require an engineered construct but relies on the target antigen being presented via the major histocompatibility complex (MHC). CAR-T based ACT can target MHC independent antigens, as well as carbohydrates and glycolipids expressed on the cell surface of tumors [14]. CD8^+^ T cells can directly eliminate tumor cells due to their cytotoxic properties, which is why they have long been considered as the most suitable T cell subset for ACT. CD8^+^ T cell-mediated killing occurs through MHC-I-dependent recognition of tumor cells and subsequent granzyme- and/or FAS ligand-dependent elimination. This also requires cross-priming by DCs as well as co-stimulation by natural killer cells and/or CD4^+^ T cells derived cytokines [15]. However, the support of innate immunity to T cell adaptive immune responses is not always present in the TME. If CD8^+^ T cells cannot be primed and activated in the TME, CD8^+^ T cell-dependent tumor elimination fails [16]. The required co-stimulation to activate CD8^+^ T cells can also be provided by CD4^+^ T cells. Current strategies aim to exploit these properties of CD4^+^ T cells in ACT [17]. CD4^+^ T cells are also suitable for ACT because they support antigen presentation from DCs, T cell homing through the secretion of chemokines such as CXCL9-11, formation of CD8^+^ T cell memory, and direct tumor elimination by granzymes, perforin, TRAIL, or FasL (reviewed in [18]). A recent study examining long-persisting anti-CD19 CAR T cells demonstrated that 9 years after therapy, the long-term protection against CD19^+^ cells was mediated almost exclusively by cytotoxic CD4^+^ CAR T cells. This suggests that beyond the short-term tumor elimination mediated by CD8^+^ T cells, CD4^+^ T cells contribute to long-term remission [19].

Because it is now accepted that the immune system shapes the initiation and progression of cancer [20], increasing efforts are being made to design and exploit adjuvants that will boost anticancer immune responses. Immune adjuvants include agonists of pattern recognition receptors, such as Toll-like receptors (TLRs), nucleotide-binding oligomerization domain (NOD)-like receptors (NLRs), Retinoic acid-inducible gene I (RIG-I)-like receptors (RLRs), C-type lectin receptors (CLRs) and cytosolic DNA sensors such as stimulator of interferon genes (STING) [21]. We will here discuss recent findings indicating that STING agonists enhance T cell anticancer responses and underscore their relevance in the context of ACT.

## STING IN CANCER IMMUNOTHERAPY

**Figure 1:**
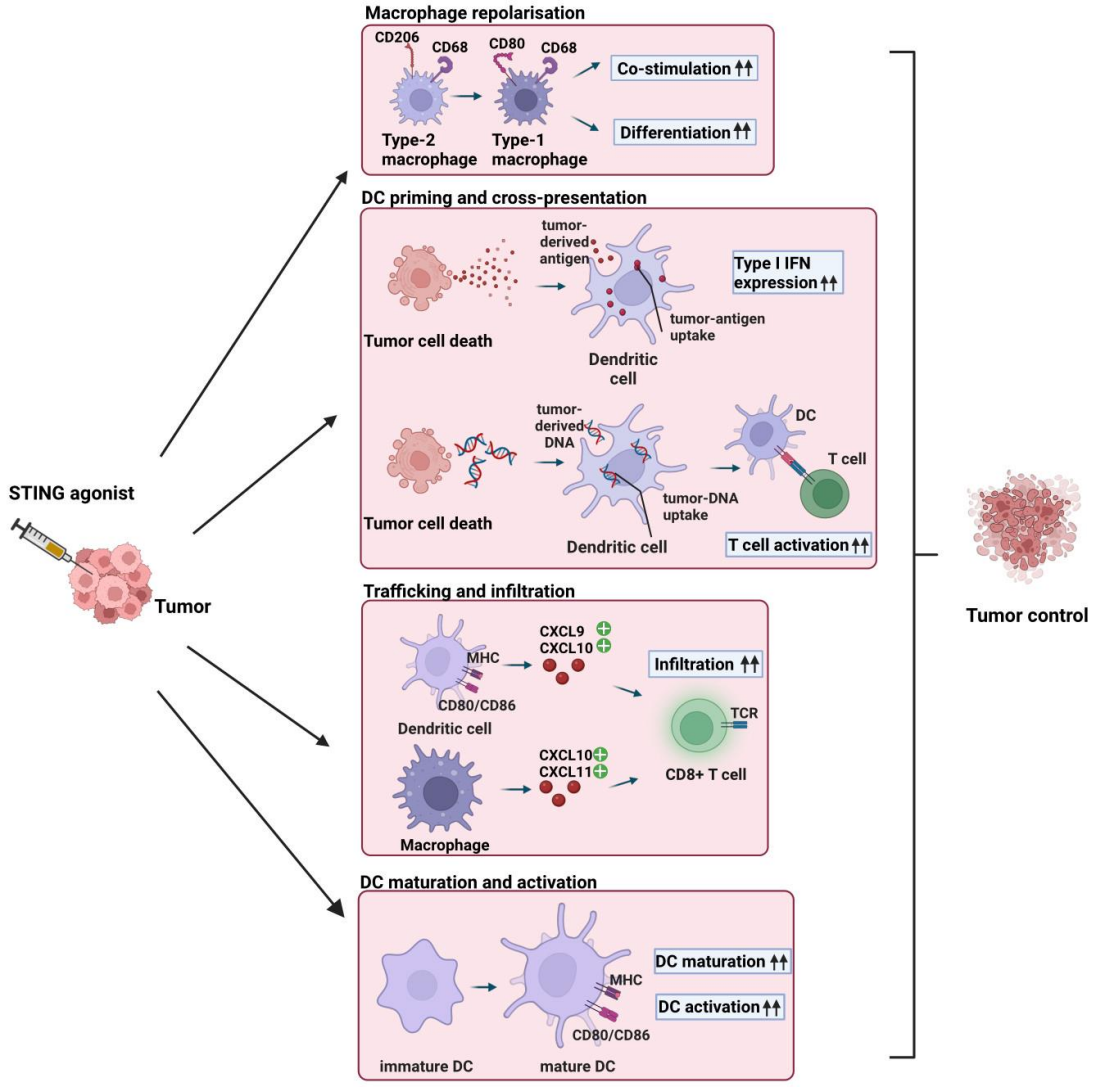
FIGURE 1: STING agonist-induced effects on innate immune cells in the TME. Intratumoral injection of STING agonists induces repolarization of type-2 macrophages into type-1 macrophages, resulting in enhanced co-stimulation and differentiation of CD4^+^ and CD8^+^ T cells [39]. STING ligand-induced tumor cell death leads to the release of tumor-derived antigens and DNA, which are taken up by DCs, resulting in the release of type I IFNs and enhanced T cell activation [23, 24, 27]. Intratumoral injection of STING agonists results in the secretion of the chemokines CXCL9, CXCL10, and CXCL10, CXCL11, which are secreted by DCs and macrophages, respectively [37, 41]. As a result, cytotoxic CD8^+^ T cells are recruited to the TME. STING agonists induces the secretion of type I IFN, leading to terminal differentiation of immature DCs and enhanced activation of DCs. Overall, intratumoral STING agonists injection leads to macrophage repolarization, DC cross-presentation, T cell trafficking, and DC maturation and activation, resulting in enhanced recruitment and activation of anti-tumor CD8^+^ T cells and improved tumor control.

The STING pathway was initially considered as a protective mechanism against intracellular pathogens through the detection of cytosolic double-stranded DNA. STING signaling is also induced following the detection of cytosolic self-DNA, which can originate from tumor cells due to genomic instability [22]. Double-stranded DNA in the cytosol is bound in a sequence-independent manner by the cGAMP synthase (cGAS), resulting in the formation of cGAMP, which acts as a second messenger and activates the STING protein in the ER. STING then undergoes conformational changes and translocates into the ER-Golgi intermediate compartment and Golgi compartment. Here, TANK-binding kinase 1 is recruited for further signal transduction and phosphorylates interferon regulatory factor 3 (IRF3). IRF3 translocates to the nucleus, where it leads to the synthesis of type I interferons (IFNs) and the activation of IFN-stimulated genes (reviewed in [22, 23]). Increased type I IFN secretion subsequently induces tumor-specific priming of T cells by antigen-presenting cells (APCs). In tumor cells, STING induction can induce cell death and thus increases the amount of tumor antigens available for APCs. This enhances the activation of T cells, their infiltration into the tumor, thereby resulting in cancer cell elimination [24]. Treatment with STING pathway agonists accordingly promotes adaptive immune responses in the TME [25]. The resulting induction of IFN-γ-expressing CD8^+^ T cells mediates anticancer responses [26, 27]. These properties support the use of STING agonists like 2′3′-cGAMP for cancer immunotherapy (**Figure 1**).

Because of STING agonist-mediated immune cell infiltration, combination therapies with ICIs were tested [28]. Promising preclinical results when combining ICIs and STING agonists were achieved in multiple cancer models, including melanoma and an HPV^+^ oral tumor [29–32]. However, these successes failed to translate into the clinic due to the pharmacokinetic properties of STING agonists that preclude effective drug delivery [33]. Furthermore, some tumors feature limited responses to STING ligands due to inhibitory mechanisms that include for instance p53 [34], ecto-nucleotide pyrophosphatases 1 (ENPP1) [35], Hypoxia-induced RNASEH2a upregulation [36], and TIM-3 [37]. To tackle some of these hurdles, a better bioavailability of STING agonists is needed. This could be achieved through more stable STING agonists or improved delivery systems (discussed in [33]).

## STING-DRIVEN ACTIVATION OF INNATE IMMUNE CELLS FAVORS T CELL ACTIVATION

Two major uses for STING activation can be contemplated. The first is to deliver STING agonists directly into the TME of the host. This approach can trigger anticancer responses [38], thereby contributing to overall tumor elimination as discussed above. Macrophages and DCs are critical innate immune cells that affect CD8^+^ T cell-mediated tumor elimination. Type-2 macrophages can be repolarized into type-1 macrophages by STING activation, which can improve the antitumor response by enhancing the co-stimulation and differentiation of CD4^+^ and CD8^+^ T cells [39]. In DCs, cGAS-STING is required for antigen presentation and cross-priming of T cells [27]. DC-mediated cross-priming is followed by recruitment of cytotoxic T cells to the TME through the chemokines CXCL9 and CXCL10 [37, 40].

Numerous studies (discussed in [24]) showed that disruption of the STING-axis led to compromised CD8^+^ T cell-mediated tumor elimination. STING activation in the TME therefore supports T cell functions. This was exploited using either CD8^+^ and CD4^+^ CAR T cells, as discussed further below.

Using a murine second-generation specific CD8^+^ CAR T cell model, the group of Sandra Hervas-Stubbs demonstrated that 2'3'-cGAMP treatment induced an endogenous T cell response, prevented antigen-loss variant outgrowth and led to an improved overall survival rate [42]. By using a bilateral B16-OVA tumor mouse model, the authors observed restrained tumor progression in the injected and opposite tumor, when the combination of 2'3'-cGAMP injection and antigen-specific CD8^+^ CAR-T cells was used. Most importantly, mice treated with the combination were the only ones to survive, while the control groups receiving standalone CAR-T cells or 2'3'-cGAMP died within the first 20-30 days.

Since the combined treatment enabled to control tumor progression in treated and untreated tumors and resulted in the survival of the mice, the authors concluded a strong synergistic effect of the STING agonist and the CAR T cells they used.

Subsequently, they investigated whether STING agonists could induce an immune response and thus could target the cancer systemically [42]. To verify this, the authors examined the presence of endogenous CD8^+^ T cells in treated and untreated tumors, lymph nodes, and peripheral blood three days after STING agonist administration. Furthermore, they analyzed the frequency of Ag-specific CD8^+^ T cells restricted to two immunodominant epitopes of B16-OVA tumors, OVA, which is a foreign antigen, and M8 is an endogenous one. Their analysis revealed a significantly increased number of M8-specific CD8^+^ T cells in the group receiving CAR T cells and administration of STING ligand in all tissues examined. The number of OVA-specific CD8^+^ T cells was generally lower, but significantly increased except in the contralateral lymph nodes. The increased presence of OVA^+^ or M8^+^ endogenous CD8^+^ T cells, particularly in the blood, lymph nodes, and untreated tumor suggests that the combination treatment induced an endogenous CD8^+^ T cell response. Overall, this may have led to the increased overall survival of the mice receiving the combined therapy. Notably, the administration of CAR T cells without STING ligand also resulted in high levels of OVA^+^ or M8^+^ endogenous CD8^+^ T cells in the untreated tumor. In line with the observations of Corrales *et al.* [40], the authors also found that cross-priming DCs are responsible for the ability of the combined therapy to eliminate tumors [42].

To next address the question of whether and to what extent the STING pathway was responsible for the observed antitumor responses, the authors used STING-deficient tumor-bearing mice or mouse STING-deficient CAR T cells. The authors found that the use of STING-deficient CAR T cells was associated with reduced antitumor effects and decreased overall survival of recipient mice but did not affect the frequency of endogenous tumor-specific T cells. In STING-deficient mice, no tumor growth delay and no increased overall survival was detected in response to the combined treatment, along with a complete lack of activated endogenous tumor-specific T cell response [42]. Therefore, the STING pathway in the host has to be fully functional for successful antitumor therapy. T cells possess an intrinsic tumor-eliminating potential, which can be triggered by STING agonist-mediated activation prior to transfer. This study by Sandra Hervas-Stubbs's group extends previous findings obtained with other STING ligands. Indeed, DMXAA and synthetic cyclic dinucleotides (CDN) elicited a systemic antitumor response dependent on STING signaling in host cells [40]. Collectively, this suggests that intratumoral injection of STING ligands can be exploited in combination with CD8^+^ T cells to target cancer systemically.

Jonathan Serody's group recently used mouse CD4^+^ T helper cells in combination with STING agonist to treat a locally advanced breast cancer model [43]. While the potential of CD4^+^ T cells for ACT is clear, the diversity of CD4^+^ T cell responses should be considered. CD4^+^ T cells are a highly heterogeneous group consisting of T_H_1, T_H_2, T_H_9, T_H_17, T_FH_, and T_Reg_ cell subsets. Each is characterized by a subset-specific cytokine profile and thereby fulfills different effector functions [44]. Although there is a large agreement that Tregs and T_H_1 cells respectively favor and restrict tumor progression, T_H_2, T_H_9 and T_H_17 cell functions in cancer are context-dependent [45]. T_H_17 cells can contrastingly affect cancer depending on their environment [46]. However, in the setting of adoptive cell therapy, IL-17-secreting T cells are considered beneficial, with reported antitumor activity in melanoma and lung cancer [47–49]. The effect of DMXAA and 2'3'-cGAMP in combination with murine Neu-specific T_C_/T_H_17 CAR T cells was investigated in the setting of breast cancer [43]. The authors have demonstrated that the combined treatment mediates antitumor control due to improved trafficking and persistence of CAR T cells in the TME [43]. T_C_/T_H_17 CAR T cells featured improved antitumor efficacy in mice receiving DMXAA, resulting in long-term control in some of the treated mice, while standalone treatments remained ineffective [43]. The infiltrating CAR-T cells were then analyzed by flow cytometry to determine the cause of the enhanced antitumor response. The authors found that some of the transferred T_C_/T_H_17 CAR-T cells had undergone a shift to T_C_/T_H_1 CAR-T cells and that DMXAA enhanced the accumulation of these T_C_/T_H_1 CAR-T cells in the TME. This phenotypic shift was accompanied by the upregulation of T_C_/T_H_1 signature transcription factors and cytokines (*Tbx21* and IFN-γ). By using anti-IFN-γ mAb, they found that tumor elimination occurred in a T_C_/T_H_1 CAR-T cell mediated manner [43]. It was previously shown that T_H_17 cells possess some levels of plasticity enabling their transformation into IFN-γ-producing T_H_1-like cells [50]. This plasticity is necessary for their antitumor response in T_H_17 ACT [51]. However, the observation that activation of the STING signaling pathway further enhances this plasticity and the antitumor responses is of high interest for cancer immunotherapy. Despite the improved effect of the combined CAR T cell and STING agonist treatment, one question remains: Why do most tumors recur as IL-17-secreting T cells are known to be long-lived and self-renewing with superior persistence [52]? To address this, the authors have performed a single-cell transcriptome sequencing of CD45^+^ cells from the TME. The authors compared leukocytes on the day of best tumor control with their counterparts collected on the day of tumor recurrence. In DMXAA treated mice, they found a shift in the myeloid cells of the TME favoring M1-like macrophages. Interestingly, their depletion by means of liposome clodronate was associated with a complete loss of the DMXAA-induced therapeutic benefit. This shift was accompanied by a stronger M1 gene expression including *nos2* and *inhba*, and a reduced expression of M2 associated genes like *retnla, mrc1, folr2*, and *il10*. Furthermore, M1-associated chemokines (*cxcl9, cxcl10*, and *ccl5*) were increasingly secreted to attract T_C_/T_H_1 T cells to the TME. In addition, they have found that while the combined therapy transiently relieves immunosuppression in the TME, this effect is short-lived and immunosuppressive myeloid cells were subsequently present in the TME. They also analyzed the T_C_/T_H_17 CAR T cells from the TME and discovered an increased expression of markers associated with dysfunction and apoptosis, providing evidence for their assumption that T cell dysfunction is the key limiting factor of the therapy. This suggested that both immunosuppression driven by myeloid cells and T cell dysfunction were responsible for the absence of complete responses to the combined therapy. To test this further, the combination of T_C_/T_H_17 CAR T cells and DMXAA was administered with anti-PD-1 and anti-GR-1 monoclonal antibodies twice a week, starting one day after the CAR T cell injection. This resulted in a marked increase in the ability of tumor-bearing mice to reject tumors. Importantly, only the combination of both antibodies was effective, indicating that both PD-L1 and myeloid cell-driven immunosuppression needed to be targeted to yield a therapeutic benefit. Next, the authors examined the effect of their T_C_/T_H_17 CAR T cells by comparing them with CAR T cells expanded with IL-7 and IL-15. The use of T_C_/T_H_17 CAR T cells resulted in an improved in vivo anticancer efficacy over the latter [43]. The authors linked this difference to the significantly higher proliferation rate of CD4^+^ CAR T cells in the spleen and the enhanced expansion of CD8^+^ CAR T cells with a central memory phenotype. Finally, the authors compared DMXAA with the other STING agonist 2'3'-cGAMP. Although the use of 2'3'-cGAMP resulted in significantly more T_C_/T_H_17 CAR T cells in the TME, there was only a minor difference in therapeutic efficacy [43]. Overall, this study suggests that STING ligands and CAR T cells have synergistic effects that can successfully fight breast cancer when combined.

## T CELL-INTRINSIC STING ACTIVATION AND ITS RELEVANCE IN ACT

**Figure 2:**
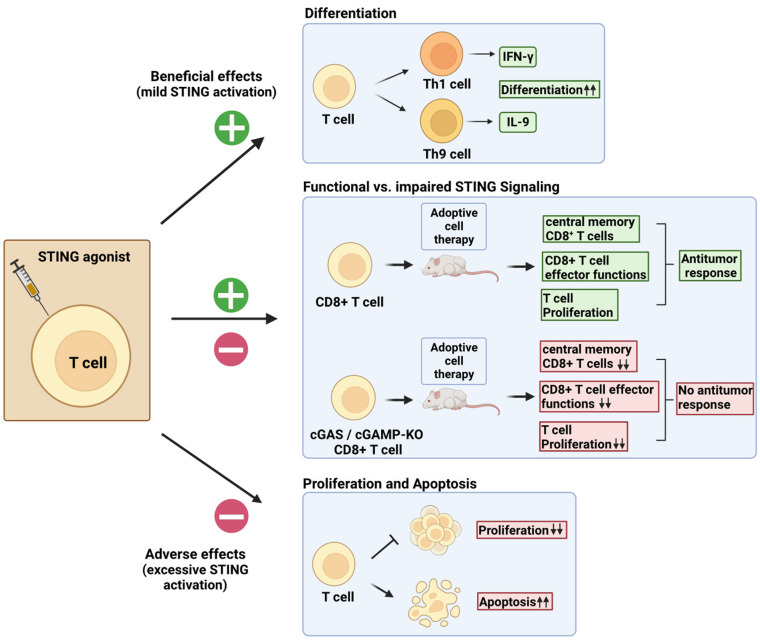
FIGURE 2: Cell-intrinsic STING activation effects on T cells. Direct stimulation of T cells by STING ligands can produce contrasting effects. Mild activation levels of STING signaling can be exploited to boost T_H_1 and T_H_9 differentiation [55]. Impaired STING signaling in T cells results in reduced frequency of central memory CD8^+^ T cells, reduced proliferation, and reduced effector functions upon adoptive transfer [56]. Potent STING signaling results in reduced proliferation and induction of cell death by apoptosis [53, 54].

Direct administration of STING agonists faces obstacles such as difficulties with drug delivery and poor pharmacokinetics. It is further accompanied by reports of autoimmune events after excessive STING stimulation [33]. To circumvent this, cells can be directly activated with the STING agonist prior to transfer. This would prevent a STING-mediated detrimental inflammatory response in the host and minimize drug-induced adverse events. We will therefore next focus on the intrinsic effect of STING agonists in T cells and discuss how STING signaling affects their viability, cell proliferation, cytokine secretion, and antitumor functions (**Figure 2**).

Alexander Poltroak's team studies on mouse T cells showed that the STING signaling pathway, already shown to be active in macrophages, was also functional in T cells. In T cells, STING induction with DMXAA leads to IFN-I production, prevention of cell proliferation, and induction of proapoptotic genes. However, these cell-adverse effects were not observable with low doses of DMXAA [53]. Andrea Ablasser's group confirmed the cell death-inducing effect of the STING agonists CMA and DMXAA, but additionally found that this was due to an intensified STING signaling response in T cells as compared to macrophages and DCs [53, 54]. It should be noted that those findings were mostly established using synthetic STING ligands such as CMA and DMXAA. By contrast, other studies reported limited apoptosis induction upon treatment of T cells with 2'3'-cGAMP [42, 55]. Liufu Deng's laboratory instead investigated whether STING signaling in CD8^+^ T cells was necessary for their antitumor effects. They found that the cGAS-STING pathway was required in CD8^+^ T cells to induce an antitumor response [56]. The use of murine tumor-specific T cells lacking either cGAS or STING showed reduced antitumor activity as compared to controls. Likewise, conditional knockout of STING in CD4^Cre+^-STING^flox/flox^ mice resulted in accelerated tumor progression in the mouse T cell lymphoma model EG7 and mouse glioblastoma model GL261. Examination of proliferative capacity and effector functions revealed that cGAS- and STING-deficient CD8^+^ T cells displayed impaired proliferation rate after adoptive transfer as compared to controls. In addition, the number of TNF-α^+^ and IFN-γ^+^ CD8^+^ T cells in tumor draining-lymph nodes was significantly reduced, suggesting dysfunctional effector functions. Finally, the authors showed that cGAS or STING deficiency in CD8^+^ T cells led to an accumulation of effector CD8^+^ T cells at the expense of self-renewing and persistent central memory CD8^+^ T cells. cGAS- or STING-deficient CD8^+^ T cells also exhibited a terminally exhausted phenotype.

Collectively, the authors' findings support the fundamental role of the cGAS-STING axis in CD8^+^ T cells for ACT [56]. Importantly, in line with the observations of other investigators [42, 55], STING treatment did not affect the cell viability of the human CD8^+^ T cells used [56]. In summary, an intact cGAS-STING axis in CD8^+^ T cells is required for their anticancer effector functions. Because excessive STING activation in T cells may trigger cell death, it remains important to carefully consider the dose of the STING ligands used to harness T cell effector functions without compromising their proliferation or viability. We have accordingly shown that STING ligands can enhance T cell effector functions and the differentiation of T_H_1 and T_H_9 cells [55].

T_H_9 cells are defined as cells lacking Foxp3 but secreting high levels of IL-9 [57]. They also secrete IL-10 and IL-21 [58, 59]. Polarization of naive T cells into T_H_9 cells is initiated by IL-4 and TGF-β and requires a complex interaction of a network of transcription factors, including IRF4, GATA3, BATF3, and PU.1 [60]. T_H_9 cells are particularly promising in the context of ACT, as Puwar and colleagues demonstrated superior antitumor properties of T_H_9 cells upon adoptive transfer as compared to other CD4^+^ subsets in a mouse model of melanoma [61]. Their superiority was subsequently independently demonstrated by multiple laboratories [58, 62, 63]. Adoptively transferred tumor Ag-specific murine T_H_9 cells were shown to provide superior antitumor immunity by eliminating variants with antigen loss [64]. All these findings provide impetus to investigating the relevance of T_H_9 cells in the ACT of cancer.

Investigations conducted by our group using different subsets of CD4^+^ T cells, including T_H_1, T_H_9, and T_H_17 cells, which were directly activated with different STING ligands, revealed that the differentiation and effector functions of T_H_1 and T_H_9 cells could be enhanced by STING activation [55].

We further demonstrated that T_H_1 and T_H_9 cells respond differently to STING ligands, as illustrated by our observation that T_H_1 cells were more sensitive to STING ligand-induced apoptosis than T_H_9 cells. STING activation enhanced human T_H_1 and T_H_9 polarization, and resulted in increased expression and secretion of IFN-γ and IL-9, the respective T_H_1 and T_H_9 signature cytokines [55]. These results showing that ligands of STING enhance T_H_9 cell differentiation are in line with published investigations indicating that pro-inflammatory components such as glucocorticoid-induced TNFR-related protein (GITR), IL-1β, TNFα, OX40L, and TL1A support T_H_9 effector functions [58, 65–68]. It is noteworthy that distinct mechanisms were contributing to the cell-intrinsic STING-driven enhancement of T_H_1 and T_H_9 differentiation. IRF3 activation was essential for the STING-mediated induction of T_H_1 cell differentiation, while mTOR signaling accounts for the increased T_H_9 cell differentiation following STING activation [55]. We tested the in vivo functions of T_H_9 cells treated with 2'3'-cGAMP in the B16-OVA melanoma model. For both subcutaneously or intravenously injected B16-OVA cells, we demonstrated that adoptively transferred 2'3'-cGAMP-stimulated tumor Ag-specific T_H_9 cells secreted more IL-9 and triggered better antitumor immunity compared to controls without STING agonists [55]. These results show that STING activation can enhance the anticancer efficacy of adoptively transferred T cells (**Figure 2**).

## CONCLUSIONS

While STING function was initially characterized in fibroblasts [69], it is now clear that STING shapes the biology of multiple immune cell types, including T cells. STING agonists have the potential to address some of the critical challenges of adoptive cell therapy. STING agonists can indeed enhance T cell infiltration and reduce tumor-induced immunosuppression [42, 43]. Despite these promising advances, the disappointing results obtained when combining STING agonists with ICIs underscore the challenge to translate the use of STING agonists into the clinic. Documented issues such as toxicity, low bioavailability, and related difficulties of administration likely prevent the clinical implementation of STING ligands against cancer (discussed in [70]). In that regard, the recent work of Jneid *et al.* that relies on the use of virus-like particles to deliver cGAMP in the TME highlights an elegant venue to circumvent some of these issues [71]. Recent results suggest that direct T cell activation by STING agonists can be exploited in the context of ACT. Both Liufu Deng's laboratory and ours have shown that T cells can be directly activated with STING agonists without triggering marked cell death [55, 56]. A thoughtful selection and careful use of STING ligands will allow harnessing of T cell anticancer functions without compromising their fitness. Further research is warranted to translate the therapeutic use of STING ligands in the setting of ACT.

## Abbreviatons:

ACT: – adoptive cell therapy,
TME: – tumor microenvironment,
STING: – stimulator of interferon genes,
DC: – dendritic cell,
ICI: – immune checkpoint inhibitors,
TILs: – tumor-infiltrating lymphocytes,
TCR: – T cell receptor,
CAR-T: – chimeric antigen receptor T cells (CAR-T),
MHC: – major histocompatibility complex,
TLRs: – Toll-like receptors,
NOD: – nucleotide-binding oligomerization domain,
NLRs: – NOD-like receptors,
RIG-I: – Retinoic acid-inducible gene I,
RLRs: – RIG-I-like receptors,
CLRs: – C-type lectin receptors,
cGAS: – cGAMP synthase,
IRF3: – interferon regulatory factor 3,
IFNs: – type I interferons,
APCs: – antigen-presenting cells.
